# Is it time we get real? A systematic review of the potential of data-driven technologies to address teachers' implicit biases

**DOI:** 10.3389/frai.2022.994967

**Published:** 2022-10-11

**Authors:** Andrea Gauthier, Saman Rizvi, Mutlu Cukurova, Manolis Mavrikis

**Affiliations:** UCL Knowledge Lab, Department of Culture, Communication and Media, IOE UCL's Faculty of Education and Society, University College London, London, United Kingdom

**Keywords:** bias, teachers, equity, artificial intelligence in education, learning analytics (LA), decision support systems

## Abstract

Data-driven technologies for education, such as artificial intelligence in education (AIEd) systems, learning analytics dashboards, open learner models, and other applications, are often created with an aspiration to help teachers make better, evidence-informed decisions in the classroom. Addressing gender, racial, and other biases inherent to data and algorithms in such applications is seen as a way to increase the responsibility of these systems and has been the focus of much of the research in the field, including systematic reviews. However, implicit biases can also be held by teachers. To the best of our knowledge, this systematic literature review is the first of its kind to investigate what kinds of teacher biases have been impacted by data-driven technologies, how or if these technologies were designed to challenge these biases, and which strategies were most effective at promoting equitable teaching behaviors and decision making. Following PRISMA guidelines, a search of five databases returned *n* = 359 records of which only *n* = 2 studies by a single research team were identified as relevant. The findings show that there is minimal evidence that data-driven technologies have been evaluated in their capacity for supporting teachers to make less biased decisions or promote equitable teaching behaviors, even though this capacity is often used as one of the core arguments for the use of data-driven technologies in education. By examining these two studies in conjunction with related studies that did not meet the eligibility criteria during the full-text review, we reveal the approaches that could play an effective role in mitigating teachers' biases, as well as ones that may perpetuate biases. We conclude by summarizing directions for future research that should seek to directly confront teachers' biases through explicit design strategies within teacher tools, to ensure that the impact of biases of both technology (including data, algorithms, models etc.) *and* teachers are minimized. We propose an extended framework to support future research and design in this area, through motivational, cognitive, and technological debiasing strategies.

## Introduction

An ambition in the field of artificial intelligence in education (AIEd), learning analytics (LA), and other data-driven technology fields is to help teachers make better, evidence-informed decisions. To increase the responsible nature of data-driven teacher tools, much attention in recent years has been paid to mitigating against racial, gender, and other biases that appear in data and algorithms that are used to support teacher decisions (see Baker and Hawn, 2021 for a recent review of the field). These biases may be eliminated to certain degrees through careful design, evaluation, and deployment of AI systems in real-world settings (Floridi and Cowls, 2019), and there is a growing interest both in research and practitioner communities focusing on responsible and ethical implementations of AI (i.e., ACM FAccT). However, education is also made inequitable by the implicit biases that teachers hold, which may lead them to grade or treat their students unfairly. There is an assumption made by designers of data-driven technologies that, by presenting teachers with “real” data and less noisy recommendations based on data, teachers are enabled to make fair decisions in the classroom (Angeli et al., 2017; Lameras and Arnab, 2022; Uttamchandani and Quick, 2022; Williamson and Kizilcec, 2022). Yet, some research has highlighted that issues around justice, equity, diversity, and inclusion are understudied in this area (Williamson and Kizilcec, 2022). To this day, it is unclear to what extent data-driven technologies more broadly, and AIEd specifically, have been evaluated in their capacity to mitigate against teachers' implicit biases, and/or if such systems have been designed with the unique purpose to target such outcomes.

This systematic literature review, which follows PRISMA guidelines (Liberati et al., 2009; Page et al., 2021), aims to investigate to what extent data-driven technologies have been evaluated in their capacity to challenge teachers' implicit biases and to document how such interventions were designed and implemented. It does this by searching for published evaluations of diverse data-driven interventions, that measured (in some capacity) change in teachers' biases or equitable teaching practices, within any educational context. This is done with a broader goal to develop a framework for designing data-driven technologies and associated research that effectively address this issue. The review asks the following research questions:

1. What specific types of teacher biases have been impacted by data-driven technology interventions?
2. What, if any, specific strategies or mechanisms underpinned these interventions?
3. How were teachers' biases or equitable teaching practices measured?
4. Which strategies were successful at transforming teachers' biases or equitable practices, and to what extent?

Answering these questions will enable us to identify clear directions and a proposed methodology and framework for future research in this area.

## Background

### Definitions

In this paper, we assert that a primary ambition of data-driven technologies in educational contexts (e.g., AIEd) is to help teachers make less biased, evidence-based decisions in the classroom. However, it is important to first define what we mean by “data-driven technologies”, “bias”, and “teachers”, as our interpretations of these terms informed the search terminology of the systematic review process undertaken.

#### Data-driven technology interventions

Firstly, we took a very broad view of data-driven technology interventions for teachers. We define such interventions as any digital technology-driven intervention that captures and processes data from a classroom or learning context (either autonomously or with human input) to produce an output intended to support teachers in making decisions that lead to more effective learning and teaching. This might include outputs from typical *AIEd interventions*, like intelligent tutoring systems, games, and other interactive platforms that implement AI, machine learning algorithms, or other modeling techniques to adapt to learners' behavior and performance (Mousavinasab et al., 2021; Tang et al., 2021; for reviews, see Lameras and Arnab, 2022). Such interventions might also include applications that do not explicitly integrate adaptive AI approaches but still process and model learning data for interpretation, e.g., through learning analytics, educational data mining, and open learner models, as described below.

*Learning analytics* (LA) typically capture learners' interactions and performance in an interactive tool, which can then be displayed visually to (i) learners themselves, so that they can self-reflect on their performance and choose new learning directions, or to (ii) teachers, so that they can adapt their teaching practices to support students or otherwise evaluate students' knowledge and skills (Kovanovic et al., 2021). These visualizations of LA might be presented to users *via* interactive LA dashboards, which promote exploration of the data, or through more traditional means, such as printouts. Rather than displaying data related to students' interactions with learning software, some visualizations might display classroom analytics, teacher/teaching analytics, or instructional analytics (Xu and Recker, 2012; Reinholz et al., 2020a); such terms may be used synonymously with LA but generally refer to interactions between teachers and students or students and their peers in physical or virtual classrooms, rather than interactions with learning software. All of these approaches create representations that are proxy models of complex teaching/learning processes and, thus, by definition, might involve biases in the representations as well as in their interpretations, which is why we considered all of them in the review.

*Educational data mining* (EDM), which focuses on finding patterns in educational data through statistical machine learning techniques, is similar in many ways to LA but arose from different origins (Papamitsiou and Economides, 2014). Another distinction is that it typically aims to leverage human judgement to enhance automated discovery and decision making, whereas LA typically takes the opposite approach, to support human decision making by leveraging automated data processes (Siemens and Baker, 2012; Liñán and Pérez, 2015).

Finally, learner models are the underlying algorithms and logic that interpret demographics, interactions, and behaviors, to make inferences about their mastery of a subject or skill, cognitive characteristics (e.g., learning styles, working memory capacity), social characteristics (e.g., culture, collaborativeness), personality traits, and learning motivations (Abyaa et al., 2019). Sometimes, these models are “opened up” to learners and/or teachers through visualizations (e.g., graphs, charts, and other images) to be scrutinized, in what is called an *open learner model* (OLM) (Bull and Kay, 2016). This visual process of opening the AI enables the viewer to reflect and make decisions about what content/training to pursue next.

#### Bias-related terms

Bias in our context is defined as “an inclination in temperament or outlook” in favor of or against things, ideas, or people, often based on “personal or unreasoned judgement” (Merriam-Webster Dictionary, 2022). Such biases can be both explicit (the person is aware of their biases and consciously acts upon them) or implicit (the person is not conscious of their biases and cannot intentionally control how these biases manifest in social perceptions and judgement formation; Greenwald and Hamilton Krieger, 2006). We will consider many types of *bias* one might hold toward diverse people, including racial, gender, dis/ability, nationality, and socioeconomic bias. There are yet other cognitive biases toward interpreting information, statistics, and data visualizations, e.g., framing effect, outcome bias, clustering illusion, and priming (Aczel et al., 2015; Valdez et al., 2018), to name only a few. Whilst we are primarily concerned with the aforementioned people-centric biases, rather than cognitive biases associated with data interpretation, we will keep the latter in mind as they may feasibly interact with the former. For instance, the framing effect bias is about drawing different decisions based on the same information that is presented in different ways (e.g., Cukurova et al., 2020)—it is feasible that this could be influenced by the outputs of a data-driven technology toward increased/decreased biases toward people. Outcome bias is about judging the quality of future decisions based on previous outcomes, which may feasibly interact with biases and prejudices against people of certain ethnic and/or socioeconomic backgrounds. Similarly, clustering illusion refers to our perceptual system noticing patterns in very sparse data, whilst priming refers to us noticing things if they are already primed in our memory. The framework by Valdez et al. (2018) lays out how diverse cognitive biases can interplay with our other human-centric biases.

In addition to bias, *fairness* and *equity* are other key terms in our search, which relate to the fair and equitable provision of education. Furthermore, the term *Equality, Diversity, and Inclusion* (EDI) may also prove relevant, as schools and higher education institutions will have EDI policies in place as safeguards for students and teachers, which may be targets for data-driven interventions (e.g., Mehta et al., 2018; Neuböck-Hubinger et al., 2020; Scott, 2020; Wolbring and Lillywhite, 2021).

#### Teacher-related terms

Finally, in this review, we specifically look for evaluations of data-driven technologies that measure changes in *teachers*' biases or equitable teaching practices. This includes *any type of teacher*, in primary or secondary school (e.g., teachers, teaching assistants), higher education (e.g., lecturers, instructors, and professors), or in informal education contexts (e.g., mentors, trainers, and tutors).

### Addressing bias in data and algorithms

The term bias generally refers to inequitable and potentially harmful outcomes, but bias can be defined in a range of ways and could be systematic or unintentional. In the context of AIEd, Baker and Hawn (2021) in their recent work summarized how potentially harmful discrimination between subgroups might arise throughout the quantitative processing at any stage in the machine learning pipeline, even at the data collection phase. Bias in AIEd literature appears typically in relation to a model's performance, which discriminates between different subgroups within data. Previous work has explored how and in what ways machine learning algorithms could be one potential source of harm to groups with characteristics vulnerable to bias, e.g., gender (Riazy et al., 2020), race and ethnicity (Hu and Rangwala, 2020), or socioeconomic status (Yu et al., 2021), based on the ways data are measured and collected, modeled in machine learning algorithms, and deployed (Kizilcec and Lee, forthcoming; Bayer et al., 2021).

It has also been pointed out in recent work that most of the sources of biases detected in the algorithms are in fact external to the algorithmic system or may develop later across the contexts (Baker and Hawn, 2021, p. 6). For instance, bias can originate at the measurement and data collection phase (e.g., historical patterns of bias in data), the model learning phase (e.g., aggregation, parameter estimation, or regulation), or the model deployment phase (e.g., a mismatch between the model development and the model use populations).

Although sources of bias that relate to the algorithmic and/or data-related biases are of significant concern, they are not within the scope of this research review. There is a growing body of research aiming to address some of these issues covered more from a technical point of view (e.g., Deho et al., 2022; Vatsalan et al., 2022). For instance, Kizilcec and Lee (forthcoming) proposed critical fairness criteria of *independence, separation* and *sufficiency* for potentially mitigating bias in the predictions of AI applications in Education. However, techno-centric solutions alone are not enough to help teachers make better and more fair decisions in their educational practice (Prinsloo et al., 2022). Contrastingly, the aim of this review distinguishes itself from the previous work in that we aim to explore how data-driven technologies have addressed *teachers*' biases, when teachers tend to show favorable behavior potentially skewed toward a particular group of students compared to other marginalized groups.

### Mechanisms of bias change

To the best of our knowledge, a framework does not yet exist which proposes how data-driven technologies can challenge implicit biases of teachers, but we can draw upon work in other fields, such as cognitive psychology and data visualization. For instance, Larrick (2004) describes prescriptive motivational, cognitive, and technological strategies for helping people make unbiased decisions, which he terms “debiasing” strategies. There is also extensive literature on how the visual and interactivity design of data visualizations can influence cognitive biases, which may interact with biases against marginalized groups. These ideas are introduced below.

#### Larrick's debiasing strategies

**Motivational strategies**. According to Larrick (2004), motivational strategies include (i) incentivising unbiased decisions and (ii) holding people accountable for their decisions. An example of incentives in the context of decision making with data is the game “Beats Empire”, which requires teens to carefully analyse trends in the (fictional) music industry to identify which artists to commission, in what genres, about what topics, etc.; players are incentivised to carefully consider the data through game rewards and achievements (Basu et al., 2020). In terms of accountability, the idea is that the potential social repercussions of an individual's decisions motivate them to engage in more careful and considered decision-making. However, both strategies require the individual to already possess the decision-making skills necessary to make unbiased decisions (Larrick, 2004).

**Cognitive strategies**. Larrick's cognitive strategies include (i) considering the opposite, (ii) training in rules, (iii) training in representations, and (iv) training in biases (Larrick, 2004). “Considering the opposite” involves training people to ask themself, “What are some reasons that my initial judgment might be wrong?” (Larrick, 2004). In this way, they might reconsider their initial impressions and engage more analytically in their decision making. “Training in rules” involves teaching people classes of decision rules, which may be achieved through relatively brief training, e.g., by working with existing examples. Previous work has shown that explicit instruction in rules, combined with practice with examples, is effective at promoting critical thinking skills and reduced bias in complex reasoning (Heijltjes et al., 2014). Analogical training is similar and involves presenting people with multiple examples of superficially different cases but then highlighting the similarities in the biases and training participants to be able to identify the analogous behaviors, which has previously been found to have long-term effect on reducing people's statistical biases (Aczel et al., 2015). “Training in representations” is about training people to translate information into a different representation (or, perhaps, to offer multiple representations) to get a different perspective on the topic (Larrick, 2004). Larrick gives the example that many people have difficulties understanding probabilities, so they could be trained to translate probabilities into frequencies, which is more intuitive to understand. Finally, “training in biases” might involve tactics such as behavioral decision therapy, which points out inconsistencies in human reasoning, thus raising awareness of potential biases (Larrick, 2004).

**Technological strategies**. Larrick's technological strategies include *statistically oriented techniques* that promote evidence-informed decision making, such as (i) linear models, multi-attribute utility analysis, and decision analysis, (ii) decision support systems, and (iii) group decision making. Larrick identifies linear models, multi-attribute utility analysis, and decision analysis as different statistical techniques that allow users to weigh several factors or attributes that might influence a decision. As discussed in Section Addressing bias in data and algorithms, these strategies may themselves be potential sources of bias, which was not addressed by Larrick back in 2004, so careful consideration should be paid to mitigating against such biases in the pipeline of their development and deployment. Larrick postulated that decision support systems build upon these statistical techniques by visualizing data-driven outcomes on displays to “facilitate information acquisition and processing” by the decision maker, as discussed further in the next section. Finally, Larrick places group decision making under “technological strategies” because making decisions as a group increases the sample size of experiences contributing to the decisions, so these decisions become more statistically sound. Larrick discusses that groups can act as an error-checking mechanism, where aberrant views are put in check, but still seeks to preserve the diverse perspectives of group members.

#### Data visualization

Data visualization is a technique frequently employed in data-driven decision support systems, and its qualities and characteristics can have significant impact on people's interpretation of the data (Brinch, 2020; Lin and Thornton, 2021). Firstly, the degree to which a data visualization matches our pre-existing schemas of common data representations will determine whether the information is interpreted quickly, using little working memory, or slowly and contemplatively, using more working memory (Padilla et al., 2018; Streeb et al., 2018; Sukumar and Metoyer, 2018). Secondly, the chosen visualization design can also bias our interpretations drawn from the data (Valdez et al., 2018). For instance, Xiong et al. (2022) demonstrated that icon arrays with either a random distribution or edge distribution led to participants frequently underestimating risk probabilities, whilst central distributions led participants to overestimate probabilities; it is plausible that such perceptual biases may then have downstream confirmatory or negatory effects on other biases (e.g., gender, race). Correll and Gleicher (2014) argue that sometimes visualizations should be purposefully embellished or distorted to facilitate understanding and/or mitigate against certain biases, though Valdez et al. (2018) argue that this is a philosophical question up for debate.

### Measuring bias change and/or equitable teaching

Self-report measures may be found unreliable when attempting to collect data about socially sensitive biases (e.g., race, gender, and disability bias), where respondents may purposefully mask their biases to avoid embarrassment, and so methods to evaluate such biases more stealthily (or implicitly) can be valuable (Gawronski and De Houwer, 2014). In response to this, Gawronski and De Houwer (2014) identified 19 *implicit* methods through which to measure a person's biases. The most notable measure is the Implicit Association Test (IAT), which assesses people's automatic associations between pairs of words (e.g., white vs. black and good vs. bad).

However, bias might also be inferred through teachers' behaviors in the classroom. Robinson et al. (2018) describe a framework for equitable teaching practices, which outlines behaviors indicative of whether a teacher is acting in an unbiased manner in the classroom. These behaviors include positioning students with an asset framing, disrupting preparatory privilege, and honoring overlapping oppression in intersectional identities. *Asset framing* occurs when a teacher acknowledges and reinforces through dialogue with students that diversity in knowledge and skills of individuals from different backgrounds is a strength that can contribute to the development of individuals and the class as a whole. *Preparatory privilege* takes place when students with socioeconomic or lifestyle advantages that prepare them more adequately for their studies are seen by teachers as naturally gifted or in some way intellectually superior to other students, which may lead to negative experiences for other students; disrupting this privilege can involve placing responsibility on the privileged student to actively engage with and bolster the participation of less privileged ones. Students may have *intersectional identities*, which relate to different aspects of their lives and can influence how they experience education and feelings of belonging toward academic communities or professional groups. Oppression within such identities—and students' concerns about whether this is happening in the classroom—should be acknowledged by teachers as legitimate and significant and not dismissed. Teachers might help address this oppression by connecting students with role models and actively promoting participation from underrepresented groups in, e.g., computer science (Robinson et al., 2018) and AI (Jagannathan and Komives, 2019), which are domains typically dominated by white males.

## Methods

Having defined our terms, discussed the role of bias in data and in people, and postulated about mechanism that might transform biases, as well as techniques to measure that change, we are now prepared to review the existing literature that evaluate data-driven interventions in their capacity to mitigate teachers' biases or support more equitable teaching practices. Please note that this review follows the guidelines for the Preferred Reporting Items for Systematic Reviews and Meta-Analyses (PRISMA), version 2020.

### Eligibility criteria

Included studies were evaluations of data-driven technology interventions that: (i) measured the effectiveness of the intervention at transforming teachers' implicit biases (e.g., gender, racial, and confirmatory) or inequitable teaching practices (e.g., as measured through change in their goals, perception, decision-making, or actions in the classroom); (ii) targeted teachers who are responsible for any age group and demographic of student (e.g., primary, secondary, tertiary, etc.); (iii) employed any type of evaluative research methodology (e.g., case study, non-randomized quasi-experiment, or randomized controlled trial, and using quantitative and/or qualitative methods); (iv) were published as scholarly outputs, e.g., thesis/dissertation, journal article, conference paper; and (v) were published in English.

We excluded evaluations of data-driven technologies that (i) did not evaluate the intervention on human outcomes related to biases or equitable teaching behaviors (e.g., only evaluated *algorithmic* or *data* accuracy and biases); (ii) were literature reviews or opinion pieces; (iii) were workshop summaries, book chapters, or whole conference proceedings; (iv) were non-scholarly publications (e.g., magazine and newspaper articles); or (v) were not published in English.

### Search method

An electronic search was performed on five databases: ACM Digital Library, IEEE Xplore, ProQuest (encompassing ERIC, IBSS, and PsycINFO), Scopus, and Web of Science. No date restrictions were placed on the search, which was performed on 15th October 2021. However, searches themselves were restricted to content in the abstract for feasibility; otherwise, the searches produced results in the tens of thousands (this is discussed in the Limitations section).

The search targeted evaluations of data-driven technologies that challenge biases of teachers in any educational context. Table 1 outlines the logic behind the search structure.

**Table 1:** General search string structure.

**Target**	**Search terms**
Data-driven technologies…	• “Artificial intelligence” **OR**
	• “AI” **OR**
	• “Educational data mining” **OR**
	• “Open learner model” **OR**
	• “Learning analytics” **OR** “classroom analytics” **OR**
	• “Teacher analytics” **OR** “teaching analytics” **OR** “instructional analytics”
	• “Data analytics” **AND**
… That challenge biases…	• Bias **OR**
	• Fairness **OR**
	• Equity **OR**
	• “Equality, diversity, and inclusion” **OR** “equality, diversity and inclusion” **AND**
… Of teachers in any	• Teacher **OR**
educational context.	• Instructor **OR**
	• Tutor **OR**
	• Trainer **OR**
	• Mentor **OR**
	• “Teaching assistant” **OR**
	• Lecturer **OR**
	• Professor

For any selected papers (see Section Included studies below), backward and forward snowballing was undertaken, to identify any additional papers that were not captured by the database searches. Backward snowballing refers to scanning the reference list of a selected paper for potentially relevant titles, checking how it was referenced in context, then looking up the full text to assess its relevance. Forward snowballing refers to searching for newer records that themselves have referenced the selected paper. For this purpose, we searched for selected papers in Google Scholar, then use the “Cited by” functionality to check the relevance of newer records.

### Selection process

Database search results were imported into EndNote v.10, where duplicates and non-scholarly and other ineligible records were removed *via* automated processing. The remaining results were then exported into an Excel spreadsheet that displayed the reference and abstract, and additional columns were added related to the eligibility criteria. Two independent coders then performed an initial screening of the titles and abstracts of all records to exclude any that did not obviously meet the inclusion criteria. Any disagreements between coders were kept at this stage for a full-text review. Full texts were then collected, and the same two coders performed a detailed review of these to determine if they matched the eligibility criteria; reasons for exclusion were documented. Using SPSS v.27, interrater reliability was assessed *via* the kappa coefficient (κ), where a value of 0.7 would be considered substantial agreement. Any disagreements between coders on records' eligibility were discussed and 100% resolved before data collection.

### Data collection and data items

The first coder modified a data-extraction spreadsheet template which had previously tested in a systematic review (Gauthier et al., 2019). After preparing the spreadsheet, both coders independently extracted information from all studies in a fully crossed design. Final categories of data in the spreadsheet included: general meta-data, study design (e.g., case study, randomized trial), study population (e.g., primary, secondary, or tertiary teachers), sample size, type of bias targeted by intervention (e.g., racial, gender, and individual), description of intervention, intervention augmentation type (e.g., tackling bias to augment teachers' goal-setting, perception, decision-making, and action—based on Holstein et al., 2020), intervention theoretical grounding and debiasing strategy used, description of study methods, description of bias-related outcome measures, description of study results, and any additional notes/observations from coders. The extractions from the two coders were compared for congruency and conglomerated to capture all relevant details of studies.

## Results

### Study selection

**Figure 1:**
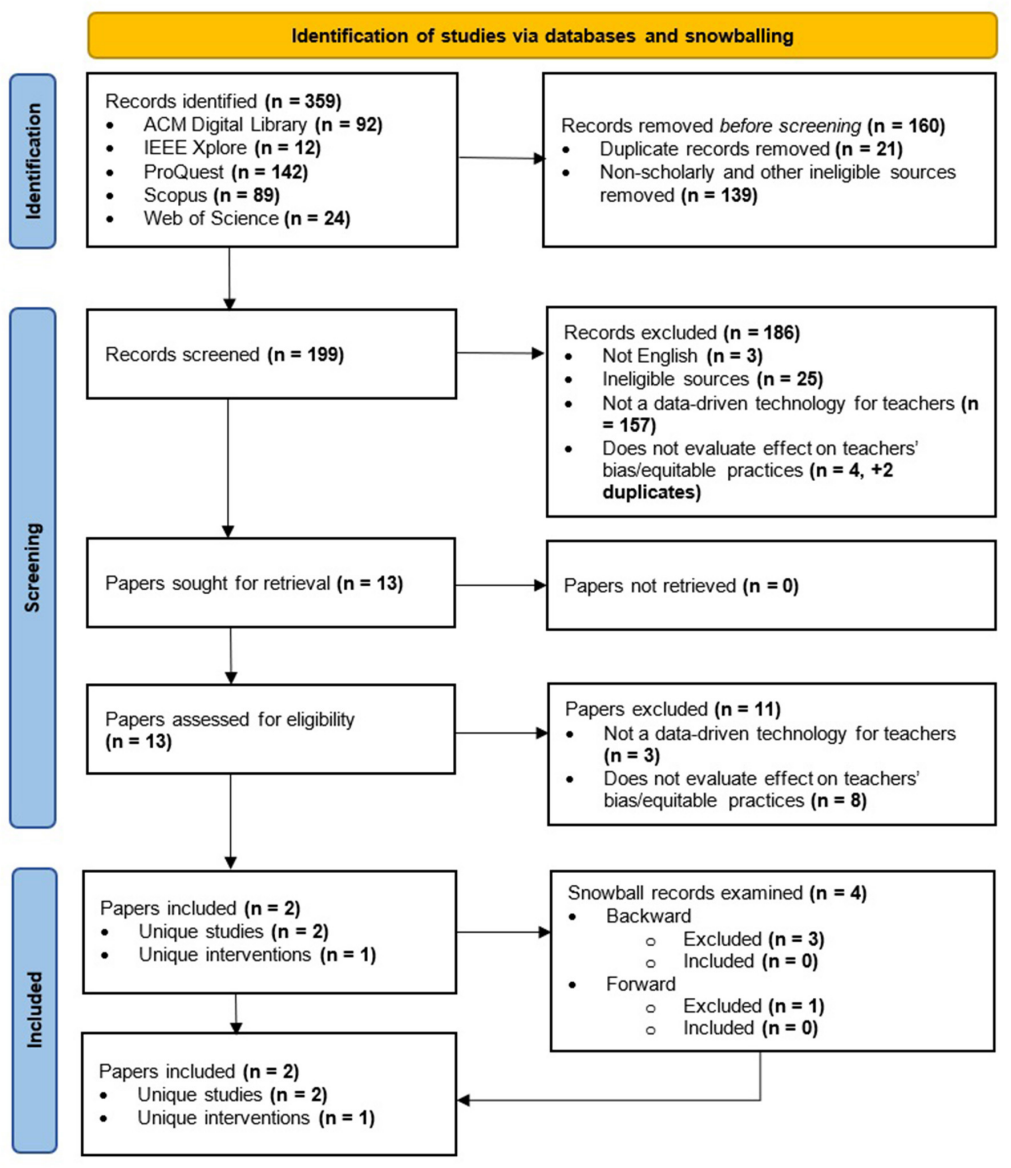
Study selection flow diagram.

A total of *n* = 359 records were identified across the five databases. Figure 1 visualizes the study selection process. Ultimately, only two papers that describe two different studies evaluating the same intervention were included. Interrater-reliability at the abstract-screening stage was very good, κ = 0.808 (SE = 0.094), *p* < 0.001, and there were no disagreements at the full-text review stage.

### Excluded studies

Due to the paucity of papers meeting the inclusion criteria, we believe that it is beneficial for further discussion to elaborate upon why so many records were excluded.

Most records reviewed during the abstract screening phase (*n* = 157) were excluded because the abstract did not discuss a data-driven technology (as defined in the Background) that targeted teachers. These excluded records were often literature reviews (e.g., Roberts and Jette, 2016; Xu, 2020), summaries of design guidelines (Vasconcelos et al., 2018; e.g., Henderson and Milman, 2019), or were about data-driven interventions targeting individuals other than teachers (e.g., Quigley et al., 2017; Tressel et al., 2020). Some of the records that did present interventions for teachers (*n* = 4) were excluded because they did not evaluate (in its broadest definition) its effect on bias/equitable teaching. These either focused on the evaluation of algorithmic bias (Whalen and Glinksi, 1976; Bogina et al., 2021), targeted the bias of the learners, rather than the teachers (Dinnar et al., 2021), or targeted teachers' biases toward particular educational techniques, such as written homework and clicker polls, rather than bias against learners (Duzhin and Gustafsson, 2018).

In the full-text review of 13 papers, 11 papers either did not report a data-driven technology for teachers or did not evaluate the effect on the intervention on teachers' biases or equitable practices. An explanation for why each item was excluded is reported in Table 2, which will factor into our discussion.

**Table 2:** Reasons for paper exclusion upon full-text review.

**Study reference**	**Data-driven technology for teachers?**	**Evaluates effect on teachers' biases/ equitable practices?**	**Explanation for exclusion**
Backer (2015)	Yes	No	Describes an AI-enhanced writing programme for students that included a dashboard for teachers to facilitate evaluation. *Whilst the abstract claimed that this could reduce bias present in traditional classrooms, its effectiveness on teachers was not evaluated*. Apart from the abstract, the rest of the paper does not discuss bias or how the intervention claims to address it.
Bailey and Michaels (2019)	No	No	Describes a decision-support system to help school administrators allocate students to classrooms. The paper does not evaluate the intervention's impact on teachers' biases and/or equitable behaviors. The intervention is aimed primarily at principals/admin who perform the allocation, though this allocation might have downstream effects on teachers' behavior in the classroom. However, *bias is only discussed in relation to how student allocation can influence students' academic performance and biased evaluation of their teachers*.
Bentivoglio (2009)	Yes	No	Describes an AI-agent, based on text and social network analysis, to evaluate the state of forum discussions in a Learning Management System. *The abstract claims that this can help mitigate instructors' subjective biases when evaluating students' progress based on forums. However, the intervention was not evaluated* and focuses on describing the algorithmic structure and performance.
Healion et al. (2017)	Yes	No	A very short paper that describes the use of a learning analytics system embedded into educational furniture to monitor the movements of students and teachers. The paper is about *using the analytics to visualize and understand human behavior and create better and more equitable, physical, collaborative learning environments*, rather than about addressing teachers' biases and teaching behaviors.
Hirose (2020)	Yes	No	This intervention aims to address bias in teachers' evaluation of students. However, it *does not attempt to impact the teachers themselves, but it changes the method of student evaluation* (e.g., from descriptive assessment, which is assessed subjectively, to multiple-choice, which is assessed objectively), so that there cannot be variation in how teachers mark students, leading to fairer assessment.
Jagannathan and Komives (2019)	No	No	Describes a course on AI delivered to girls, to increase the participation of females in the AI field in the future. It is not an intervention intended to impact teachers, though it could be delivered by teachers. *Not relevant to the current review*.
Nguyen et al. (2018)	Yes	No	Evaluates the usability and usefulness of a learning analytics dashboard that accompanies a university's learning management system, to help instructors track learners' engagement with recorded video lectures. Is not explicitly designed to or evaluated for its capacity address bias or fair teaching practices, but *participants reflected how it might inadvertently negatively bias teachers' behaviors*.
Pei and Xing (2021)	Yes	No	Aims to visualize the machine-learning pipeline to help teachers spot at-risk students. The “fairness” component is in relation to personalizing interventions for at-risk students and not allowing their demographics to bias this algorithmic personalisation. As such, *the focus is on algorithmic, not teacher, bias*.
Perez Gama et al. (2017)	No	No	Reports a model for peaceful and equitable social and educational reform—and tools to facilitate this reform—in Colombia. *Not relevant to the current review*.
Shettar et al. (2020)	Yes	No	Aims to create fairer evaluation of collaborative group work through analytics that accurately reflect each individuals' effort. This intervention does not attempt to target instructors' biased evaluation directly, though there is potential for it to indirectly impact biased behavior (for better or for worse). However, *there is no evaluative component to the paper*.
Srivastava et al. (2021)	Yes	No	*Aims to address equitable practices but not from the point of view of changing teachers' behaviors*; the technology facilitates communication between differently abled students and teachers to allow students to be in the same classroom as other children (the teachers are not choosing to have differently abled children removed from/included in the classroom). The intervention itself is *not evaluated* in this paper.

### Included studies

#### General characteristics of studies

The two included studies were reported by the same first author and evaluated the same data-driven technology intervention through case-study methodologies. The first study (Reinholz et al., 2020a) included *n* = 3 Mathematics instructors from a minority-serving higher education institute in the USA. The second study (Reinholz et al., 2020b) included *n* = 6 STEM and non-STEM instructors from (presumably) the same institution as the first. The case studies took place over the course of a full academic semester.

#### What specific types of biases have been impacted by data-driven technologies?

The intervention was geared toward higher education instructors to increase *participatory equity* in university classrooms, which refers to providing all students with fair opportunities to participate in classroom activities and discussions. To increase participatory equity, the intervention targeted gender, racial, and dis/ability biases, though the studies focused on gender and race due to the rarity of students presenting openly with specific disabilities.

#### What specific strategies or mechanisms underpinned these interventions?

The intervention itself was a professional learning community that held meetings approximately every 2 weeks, with the aim of promoting equitable teaching practices. In short, the intervention targeted teachers' biases through a combination of explicit training, data visualization of classroom analytics, and a learning community that held debriefing sessions.

**Explicit training**. The first two meetings were 1-h *training sessions*, which covered the analytics software, as well as training on implicit bias, microaggressions, and cultural competence.

**Data visualization**. Teachers were asked to video-record interactions in their classroom. The EQUIP software was used by the researchers to code the recordings in units of verbal interaction between instructor and students. The coded output was then presented to instructors as an interactive *visual analytics* report, with interactions between instructor and student disaggregated by race and gender in histograms, line charts, and heat maps; the idea is that this would enable instructors to identify whether they were facilitating participation in one group of students (e.g., male, white) over other typically marginalized groups. The EQUIP software is a data-driven technology but does not currently contain any AI or machine learning. The software is accessible at: https://www.equip.ninja/.

**Learning community**. Following a constructivist learning paradigm, the professional learning community engaged in several sessions of collaborative reflection, where they discussed the EQUIP visual analytics and focused on small actionable changes that the instructors could make to improve participatory equity, in what the authors term “debriefing sessions”. In total, the instructors in the learning community engaged in four (2020a) to five (2020b) debriefing sessions, wherein they discussed data from one or more video-recorded lessons in the weeks that preceded. It should be noted that the video recordings were made in physical classrooms in Reinholz et al. (2020a) but, in Reinholz et al. (2020b), due to the COVID-19 pandemic, the first two recordings were made in physical classrooms, whilst the last three were made in through recording virtual classroom sessions (e.g., in Zoom). These debriefing sessions also moved online at this time in the 2020b study and the discussion in these sessions began targeting concepts related to equitable practices in *virtual* classrooms.

Furthermore, Reinholz et al. (2020a) describes how the combination of visual analytics and debriefing sessions followed four principles of good feedback: non-evaluative, supportive, specific, and timely which are overlapping with good practice in formative feedback in general (Shute, 2008). The visual analytics were *specific*, in that the data was disaggregated by different bias-related factors, but also *non-evaluative* because they reserved judgement about whether biased behaviors could be inferred from the analytics. The debriefing sessions were *supportive*, giving instructors a safe place to work toward common goals of equitable teaching. Finally, the feedback delivered through both these mechanisms was *timely*, in that it was delivered throughout the semester, enabling the participants time to engage in retrospective reflection and plan how they would act upon the feedback.

#### How were teachers' biases or equitable practices measured?

Both studies used pre- and post-intervention qualitative methods to measure bias and equitable teaching practices. Reinholz et al. (2020a) involved pre- and post-intervention *interviews* with participants, focusing on teaching experience, conceptions of equity, specific equitable teaching practices, interpretation of EQUIP analytics, and experiences in the learning community. Reinholz et al. (2020b) involved pre- and post-intervention *surveys* with participants measuring similar constructs to Reinholz et al. (2020a).

Both studies also used quantitative measures (the participation analytics) generated by the intervention itself. As described in the previous section, the visual analytics generated by the EQUIP software required researchers to code instances of instructors' interactions with students in the classroom. This data was used not only as part of the intervention, but also to track changes in instructors' equitable teaching behaviors over time; such changes were reported qualitatively. Whilst researchers coded these videos, they also made notes about interactions, which were used to qualitatively assess the intervention. In addition, in 2020b, the authors discussed these notes with participants during the debriefing sessions to get a more complete picture of the success of the intervention.

#### Which strategies were successful at transforming teachers' biases or equitable practices, and to what extent?

Because only one intervention was found eligible in this review, we cannot make a comparison between different strategies. However, the EQUIP-facilitated intervention showed evidence of effectiveness in both case studies.

In Reinholz et al. (2020a), two instructors were able to increase the overall classroom participation over the course of the semester, by calling on a greater diversity of students (the third instructor only had data collected during a single session). One female instructor realized that she allowed male students to dominate classroom conversations, so she started calling on female students more frequently. One male instructor claimed the intervention helped him realize a group of Filipino students were not participating and that he paid more attention to these students outside of the monitored sessions. More generally, participants described several benefits to the intervention: (i) the visual analytics brought awareness to their own (un)equitable behaviors, (ii) the group discussions helped them formulate concrete ways of improving practice (one participant suggested that this was more valuable than the data itself), (iii) the intervention as a whole helped them move from thinking about equity to actually re-evaluating their teaching practices, and (iv) the timeliness of the sessions allowed them enough time to action the feedback and change their behaviors. However, some disadvantages were noted: (i) participants found it awkward to discuss racial dynamics in group sessions—and this was experienced differently for white and non-white participants, and (ii) one participant noted that it was difficult to change behaviors and stick to those changes within the course of a single semester.

In Reinholz et al. (2020b), instructors noted a decrease in student participation when moving online; there were no consistent patterns based on gender or race to suggest that one demographic of individuals may have been more negatively impacted than others. Overall students' participation in online sessions increased over time. Adopting multiple forms of online participation (e.g., chats, breakout rooms) in addition to full-class discussions were thought to increase females' participation in one classroom, whilst there were no notable disparities in the other five classrooms. The results for racial minorities were also mixed, showing that minorities' participation increased in one class with the introduction of chats and breakout rooms, whist in others, minorities participated more in full-class discussions. The intervention enabled the instructors to identify (and later apply) key practices to promote equitable teaching in online environments, such as creating an inclusive curriculum (e.g., by connecting the curriculum directly to students' lived experiences) and using different forms of participation that may be more appealing to different groups (e.g., polling, breakout groups). The authors discuss similar benefits of the intervention in terms of mitigating implicit biases and promoting equitable teaching practices, as 2020a.

## Discussion

### There is not enough evidence to show that data-driven technologies help teachers make less biased decisions

A core premise of research on data-driven technologies for teachers is that they enable teachers to engage in fairer and more equitable decision making and teaching practices (Angeli et al., 2017; Lameras and Arnab, 2022; Uttamchandani and Quick, 2022; Williamson and Kizilcec, 2022). Whilst much literature has demonstrated that biases can potentially be addressed *algorithmically* (e.g., Pei and Xing, 2021, from this review), here we question the extent to which this automatically leads to less biased decisions by teachers.

Based on the findings of this review, this argument requires further evidencing. Through our search of the literature, we found some papers (e.g., Bentivoglio, 2009; Backer, 2015; Shettar et al., 2020) that claimed their interventions would positively impact on teachers' biases or fair teaching practices, but did not measure or assess this in anyway. Several other papers looked at improving equity in classrooms but from an angle other than targeting teachers' biases, such as getting more girls involved in AIEd (Jagannathan and Komives, 2019), removing subjective assessments of students (Healion et al., 2017), and facilitating communication between teachers and disabled students (Srivastava et al., 2021).

*Only two papers*, published by the same research group, evaluated the effectiveness of an intervention for mitigating teachers' implicit biases (Reinholz et al., 2020a,b). It should be reiterated that we *did not* aim to select only interventions created with an explicit goal of reducing bias, as this one did. We aimed to select research that *explicitly measured* the impact of the intervention on teacher's biases, in its broadest sense. It just so happened that the only two papers that measured bias in teachers involved an intervention that was explicitly designed to reduce such bias. This focus on explicitly mitigating teacher biases is important, especially given that one of the excluded papers from our search found, by happenstance, that their data-driven intervention could potentially exacerbate bias and unfairness (Nguyen et al., 2018). Similar findings are found in the area of behavior management; ClassDojo is a popular classroom behavioral management system that allows teachers to reward or punish children for good or bad behaviors; parents and school staff can view visualizations of children's data to see how they are behaving in the classroom (Williamson, 2017; Lu et al., 2021). Lu et al. (2021) found that, by explicitly codifying and simplifying children's complex behaviors as either bad or good within teachers' specific sociocultural constructs, teachers were found to have a bias toward prosecuting the behaviors of children from specific sociocultural backgrounds—particularly young black boys. Further onto this, by making this biased behavioral data available to other stakeholders, e.g., headteachers, the reputations of these children for being “bad” was perpetuated, exacerbating existing inequalities in children's education, and decreasing their motivation and engagement in schooling. Whilst ClassDojo is a behavior-management tool and the visualizations are not intended for teacher decision making and, thus, was not identified in our review, it is plausible that a similar negative effect might be observed with other data-driven technologies for educational decision making. Related examples are found in the data-driven “educational surveillance” literature (mostly in higher education), which evaluate interventions that track students' interactions in educational platforms and report or visualize this data to teachers in some way. For instance, Rabbany et al. (2012), Farrell et al. (2013), and Lam et al. (2019) all discuss different LA dashboards that enable teachers to make “fairer” evaluations of students' participation in online forums and group activities, by “identifying the “workers and the lurkers”” (Rabbany et al., 2012) or the “free riders” (Lam et al., 2019) in the class. The idea is that such approaches help teachers understand which students are not contributing and can adapt their grades accordingly. Whilst these authors do not evaluate the “fairness” of any resulting decisions, this approach might exacerbate possible issues of preparatory privilege (Robinson et al., 2018) that may be leading to inequity in participation in the first place. It might further provoke unconscious biases in teachers toward non-participants in future interactions, as also noted by Nguyen et al. (2018).

Given that some research has demonstrated inadvertent negative effects of data-driven technology on teachers' biases (Nguyen et al., 2018; Lu et al., 2021), we argue that *now is the time to start explicitly and purposefully designing our interventions both to mitigate against worsening biases and to actively challenge existing biases*.

### Approaches to the design and evaluation of data-driven technologies to challenge teachers' implicit biases

The included studies by Reinholz et al. (2020a,b), as well as their associated publications (Sukumar et al., 2020; Reinholz et al., 2021), are a good starting point toward the above call to action. Not only were these studies explicitly designed to mitigate teachers' implicit biases, but they measured the impact of the intervention. Their results indicate that a multidimensional, socio-technological approach to debiasing may be effective. They also give us insights into other considerations for transforming teachers' behaviors and measuring these behaviors in future research, as discussed below.

#### A multidimensional, socio-technological approach

As might be expected from a data-driven intervention, what Larrick might term a technological strategy was the first dimension of EQUIP in the form of a decision support system that presented statistics to teachers on their behaviors, and which was supported by data visualization of the classroom analytics. Both papers by Reinholz et al. conclude that the analytics helped teachers view their classrooms and their practices in a new way. However, it is unclear to what extent the format and design of the visual analytics, as well as their interactivity, influenced this effect. As the research team acknowledged in Sukumar et al. (2020), data visualizations should be “rigorously validated before they are used in practice”, but the design of these elements were not explicitly assessed to our knowledge. Given that the design features of data visualizations can bias our interpretations of data (Padilla et al., 2018; Streeb et al., 2018; Sukumar and Metoyer, 2018; Valdez et al., 2018; Xiong et al., 2022), future research and development of visual analytics might consider how our perceptual system can interact with other biases we may hold (e.g., by consulting the framework by Valdez et al., 2018), and thereby design data visualizations in such a way to control for this as much as possible.

The learning community was a second dimension of EQUIP, which was instrumental in helping teachers identify areas of personal improvement in their equitable practices and stay accountable to others in the community, which is supported by previous literature around social sensemaking (Maitlis, 2005; Puussaar et al., 2017). Social sensemaking in the context of data-driven technologies might involve sharing and discussing data with others. This has been shown to facilitate meaningful comparisons that led to better understandings of baselines or “normal” data in a personal health-data context (Puussaar et al., 2017), and bringing people together as part of a community with shared values and goals (Maitlis, 2005). Social sensemaking as a debiasing strategy aligns with Larrick's technological strategy of *group decision making* and his motivational strategy of *being accountable to others*. That said, it was noted that some teachers found it “awkward” to discuss some topics, particularly race, in a group setting, and that persons of color could potentially feel unsafe around individuals making “color-blind” comments (Reinholz et al., 2020a). Even so, research suggests that open discussion around such issues remains an important strategy in overcoming systemic discrimination (Harries, 2014; Song, 2018).

Finally, a third dimension to the EQUIP-based intervention was the formal bias pre-training (similar to the *cognitive strategy* proposed by Larrick, 2004), to make teachers explicitly aware of their implicit biases in the first place. It is unclear what role this initial strategy played in the effectiveness of the broader socio-technological intervention.

Despite the effectiveness of the intervention, it is unclear if such a multidimensional approach is sustainable and scalable. The EQUIP tool is available for teachers to use independently (www.equip.ninja), outside the context of a community of practice, so future research might investigate the use of the technological strategy on its own in comparison to its use with the additional social and bias-training components, to determine the independent and cumulative impact of these different strategies.

#### Other considerations for transforming habits

Whilst Reinholz et al.' intervention took place over an entire academic term, offering the teachers timely feedback and the ability to reflect and put reflections into practice, they still expressed that this was a relatively short amount of time for them to change their behaviors and adhere to those changes. As Bourdieu (2000, p.4430) states, “Habitus is not destiny; but symbolic action cannot, on its own, without transformation of the conditions of the production and transformation of dispositions, extirpate bodily beliefs, which are passions and drives that remain totally indifferent to the injunctions or condemnations of humanistic universalism (itself, moreover, rooted in dispositions and beliefs)”, which emphasizes the difficulties in transforming people's engrained habits and implicit beliefs. Similar results of regressing back to “normal behaviors” were also observed in semester-long analytics visualization interventions for students (e.g., Zhou et al., 2021). As such, future research should seek to measure the effects of data-driven technologies on teaching behaviors over an extended period, to allow time for change to become integrated into teachers' habitus and sustained.

Ultimately, the specific participants in the two studies by Reinholz et al. were already interested and invested in equitable teaching practices, so it puts into question whether the approaches to bias transformation used in the intervention would be effective for teachers without this keen and explicit interest. Hence, data-driven interventions should seek to make teachers' implicit beliefs explicit to them (*via* a method more engaging than bias training), to leverage this as a potential source of motivation for change. For instance, future research might draw upon work in *data journalism* and *data storytelling*, which has been successful in provoking cognitive dissonance (and, possibly, change in beliefs) in readers, to provoke motivation for change. Storytelling has been shown to bias our sensory perceptions and opinions of stimuli (e.g., Skov and Pérez-Cueto, 2015), as well as influence our beliefs, attitudes, and treatment toward marginalized groups (e.g., Long et al., 2022). Storytelling elements that accompany data (e.g., adding narratives to critical data points, adding shaded areas to cluster information, highlighting areas of the visualization with different colors, emphasizing key points and trends, and adding a prescriptive title to provide a straightforward insight about the data) has been found to support sensemaking of data visualizations (Echeverria et al., 2017). In particular, narrative has been found to increase the perceived intensity of data trends, increase viewer's trust in the information, and increase their emotional reaction to data (Braga and Silva, 2021), which may be linked to motivation. In the domain of data journalism, data visualizations juxtaposed with textual narratives about common misperceptions (e.g., about migrants) was found to reduce misperceptions of readers with low prior knowledge by comparing real data about migrants to data about the public's assumptions about migrants; though, it should be noted that the intervention was ineffective for readers with strong prior beliefs about the topic (Mena, 2021). Nguyen et al. (2019) is developing a similar strategy in data journalism that elicits news readers' beliefs and misconceptions about controversial topics through interactive visualizations that compares each readers' predictions to the “real” data, thereby provoking cognitive dissonance in the reader and (possibly) conceptual change. However, this strategy is unexplored within the domain of data-driven technologies to support teacher decision making.

#### Measuring change in biases and teaching practices

A strength of the studies by Reinholz et al. was the way in which change in equitable teaching and decision making was evaluated. They leveraged videos of teachers' actual practices in the classroom to measure real-world change over time, which is a good alternative to traditional approaches of measuring bias (for examples, see list by Gawronski and De Houwer, 2014). Some of the practices enabled by EQUIP, such as connecting the curriculum directly to students' lived experiences, relate directly to honoring intersectional identities described by Robinson et al. (2018)'s framework for equitable teaching practices. However, it is unclear if promoting equal participation from minority groups goes far enough—whilst this is a good starting point, future research might explore how data-driven strategies can help teachers position students with an asset framing, disrupt preparatory privilege, and honor overlapping oppression in intersectional identities (Robinson et al., 2018).

### Toward a conceptual framework for debiasing with data-driven technologies

We offer below a conceptual framework to guide the design of future data-driven technologies, based on the discussion above and Larrick (2004)'s motivational, cognitive, and technological debiasing strategies. We have re-framed Larrick's framework under the central theme of *technological strategies*, contextualized around *data-driven decision support systems*, and extended *by the inclusion of social, emotional, and data visualization strategies*. We argue that the way decision support systems are designed (e.g., through data visualizations and other perceptual and/or narrative components) and implemented (e.g., in a social context), should take into account these strategies, and we offer examples based on work in the field suggesting that this is feasible. An explanation of the framework is followed by suggestions on how it should be used and the next steps toward validation of the framework.

#### Explanation of the framework

**Figure 2:**
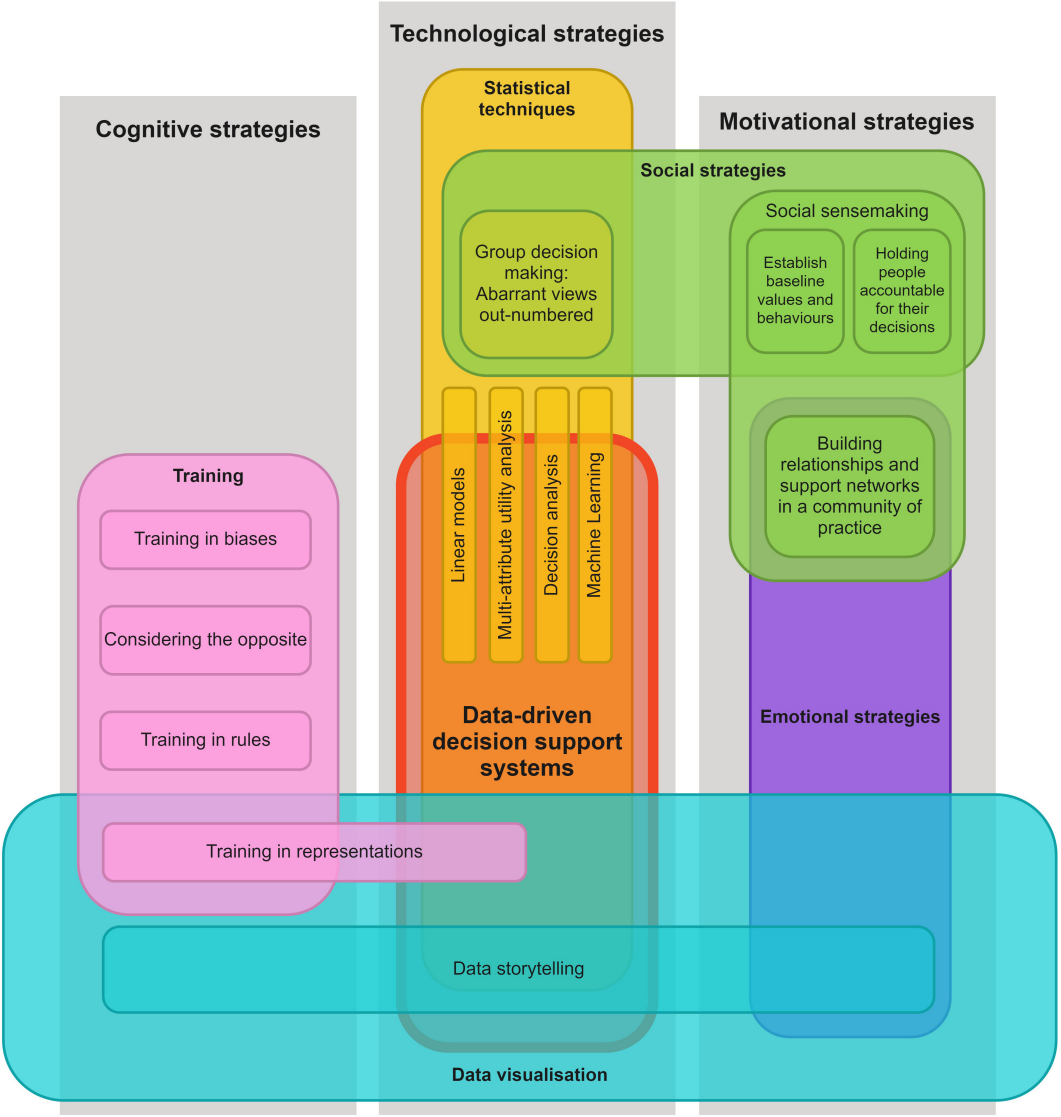
Debiasing with data-driven technologies framework.

Our framework (Figure 2) leverages Larrick (2004) three categories of debiasing strategies (represented in gray columns), but it places *data-driven decision support systems* (a technological strategy) at the center of the framework (represented by the red block). Features that overlap with this block are strategies that might be integrated into such a technology as a debiasing strategy. For instance, Larrick states that statistical strategies (e.g., linear models, multi-attribute utility analysis, and decision analysis) could feed into a decision support system, which is visualized by the thin yellow blocks; we have added other modern machine learning techniques to this list (e.g., LASSO, SMOTE, SHAP, and LIME). These specific statistical strategies also extend beyond the red block, which represents that they can be used outside the context of a decision support system, as a stand-alone strategy. Similarly, several other strategies do not overlap with the red block whatsoever, which suggests that these are external strategies that could be implemented *in conjunction* with a decision support system, like social sensemaking (in green) and bias training (in pink), as was identified in Reinholz et al.' multidimensional approach.

Another modification of Larrick's framework is that we recontextualised group-centered strategies under the sub-categorization of social strategies (green blocks), which spans the technological (i.e., group decision making) and motivational (i.e., holding people accountable to others) approaches already proposed by Larrick. However, it is extended with social sensemaking strategies (i.e., establishing baseline values and behaviors, building relationships and support networks), inspired by the findings of this review. For instance, teachers may be reluctant to accept AI-based data-driven technology recommendations when they contradict their previous knowledge about their students due to their confirmation bias (Nazaretsky et al., 2021). However, presenting teachers with some explanations of (i) how data-driven technologies make predictions, particularly compared to the human experts, (ii) how they can complement and give additional strengths to teachers, rather than replacing them, and then (iii) allowing them to discuss this in groups, can indeed reduce some of the biases of teachers, particularly within social sensemaking contexts (Nazaretsky et al., 2022). Furthermore, this model recognizes that aspects of social sensemaking can play on emotions as a motivation for debiasing (represented by the addition of *emotional strategies*, in purple). It should be noted that *incentives* as a strategy was left off this model, since is unclear how this might manifest in real-world decision-making tools, especially for teachers, where explicitly incentivising fair behavior toward children may be inappropriate or lead to undesirable results.

A prominent extension of the framework is the addition of *data visualization* strategies (in cyan-colored blocks), which cuts across technological, cognitive, and motivational categories. Data visualization is technological strategy because, as discussed in the Background section, it is an integral component to many data-driven technologies, e.g., through learning analytics displays or open learner modeling techniques (Bull and Kay, 2016; Conati et al., 2018). It might also be considered a cognitive strategy because the visual qualities and characteristics of data visualizations can draw our attention toward some aspects of data over others (Brinch, 2020; Lin and Thornton, 2021), influence the speed at which we think (Padilla et al., 2018; Streeb et al., 2018; Sukumar and Metoyer, 2018), bias our interpretations of the data (Valdez et al., 2018; Xiong et al., 2022), and can attempt to offer an objective view of learning and teaching behavior (i.e., it can be non-evaluative, as Reinholz et al. suggest, though attention must be paid to ensure the method of data collection is unbiased as well). Additionally, it might be considered a motivational strategy—particularly a source of emotional motivation (purple block)—through the use of *data-storytelling* techniques that can evoke empathetic reactions from viewers (Nguyen et al., 2019; Braga and Silva, 2021; Mena, 2021; Lund, 2022). As discussed in the previous section, since data storytelling techniques can also facilitate sensemaking of data visualizations (Echeverria et al., 2017), it might also be considered a cognitive strategy. Moreover, through participatory and inquiry-driven design of data visualizations with teachers (e.g., Pozdniakov et al., 2022), teacher biases can be made explicit and potentially addressed in a safe and supportive social environment *before* they are fed into to the final data visualizations and narratives.

Finally, we have extended Larrick's cognitive training strategy, *training in representations*, to overlap with data visualization because people's ability to translate between visual representations may enable them to view data from different perspectives and make alternate interpretations, which may feasibly impact their choices and behaviors (Zuk and Carpendale, 2007; Parsons, 2018). It also extends into the main data-driven decision support system and statistical techniques blocks, since such training and use of different visual representations could be integrated directly into a data-driven teacher tool, e.g., using explainable AI and open learner modeling techniques (Bull and Kay, 2016; Biran and Cotton, 2017; Conati et al., 2018). The other training strategies proposed by Larrick may also be relevant to the teacher decision-making context, such as *considering the opposite*; this strategy may be analogous to inhibitory control or “stop and think” training, which has been found to help people suppress their initial, intuitive reactions to stimuli and engage in more analytical thinking (Mareschal, 2016; Bell et al., 2021; Mason and Zaccoletti, 2021).

#### How to use the framework

We envision that this framework will be used, firstly, by designers and researchers *during the process of designing and evaluating* data-driven technologies that support decision making. Specifically, they can use it as a quick visual reference for what types of strategies might be integrated directly into their technology or be used as external, complementary strategies, to promote debiasing; this can be used in conjunction with the description of the framework above, to acquire indicative examples of how and why these strategies have been used by others and determine if they are worth incorporating into the intervention. We encourage robust documentation of which strategies have been incorporated and evaluation of how these have impacted users' biases, which may support a future meta-analysis, lead to replicability, or simply refinement of the framework (see Next steps, below).

**Figure 3:**
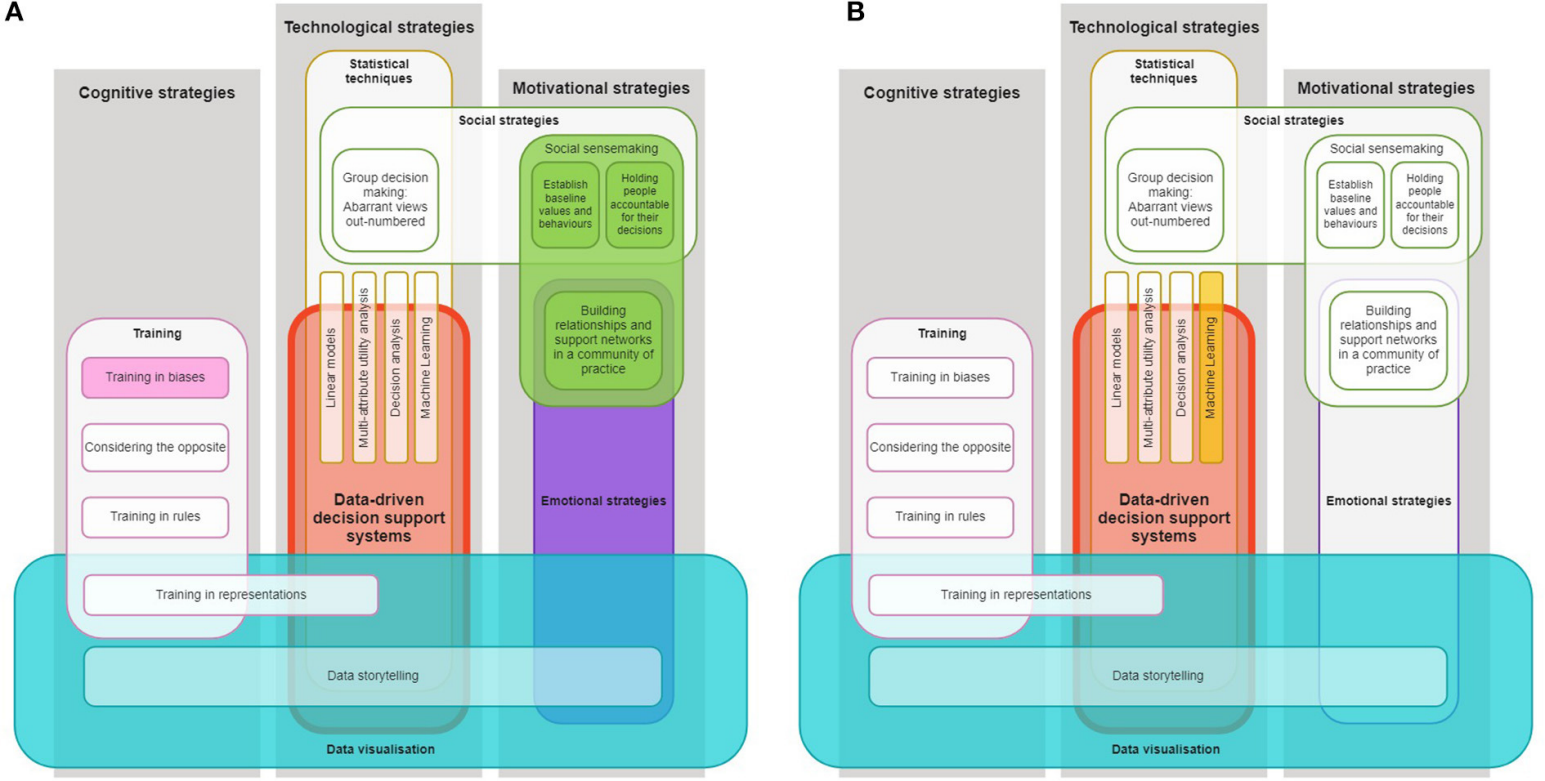
Debiasing with data-driven technologies framework as applied to **(A)** the EQUIP intervention (Reinholz et al., 2020a,b) and **(B)** an intervention to identify at-risk students (Pei and Xing, 2021). Only strategies represented by color-filled blocks are applied in these interventions.

Secondly, the framework could be used by researchers to analyse existing data-driven technology interventions and estimate their potential for debiasing. Doing so may highlight where these interventions are deficient, which may lead to the further improvement of the intervention. For example, Figure 3A shows the debiasing framework as applied the EQUIP intervention, wherein strategies that were not used are whited-out. This might lead researchers to identify that data storytelling and/or some statistical techniques could potentially improve the debiasing capacity of the EQUIP intervention. The framework can similarly be used to analyse interventions that were not designed to explicitly promote debiasing. For example, Figure 3B shows the framework as applied to Pei and Xing (2021)'s intervention that help instructors identify students at risk of dropping out, through the use of machine learning techniques and complex and varied data visualizations. Researchers might consider whether, e.g., training in representations, could support instructors in making less biased decisions based on these varied visualizations. Researchers could then proceed with an assessment of how changes of the intervention impact on users' biases, again informing our understanding of the relevance of these strategies in specific contexts.

#### Next steps

The conceptual framework described above is but a starting point to inspire the consideration of debiasing strategies during the design and evaluation of data-driven technologies for decision making, particularly as it applies to a teaching context. However, its road to validation will be a long one. Validation will occur incrementally through active participation of the research community who should begin measuring bias and/or equitable practices during the assessment of their interventions, and documenting the debiasing strategies that they employed, if any. We need to build up the evidence base of how effective these different strategies (in isolation and in combination) are in practice and then modify and refine the framework as new evidence is generated.

## Limitations

Whilst this systematic review followed established best practices with PRISMA guidelines, it suffered from a few limitations. Firstly, we limited our database searches to the abstracts of records because terms related to artificial intelligence, analytics, and bias return tens of thousands of results, which would make the review unfeasible. Although we cannot exclude the possibility that papers might evaluate interventions' impact on teachers' bias in their results sections, but do not report this in their abstract, since we set out to select research that explicitly measured the impact of the intervention on teacher's biases, we think it is realistic to expect this would be mentioned in the abstract. Nevertheless, we feel that it is unlikely that this decision missed out on many relevant papers, for two additional reasons: (i) papers that meaningfully acknowledge and empirically measure teachers' bias are likely to highlight this upfront in their abstracts, since this is a “hot topic” that would aid with discoverability and add novelty and relevance to their work; and (ii) we also performed a backward and snowballing approach on our included studies, which involved looking at what research they (as experts) regard as relevant to this domain, as well as looking at who has referenced them. This second point especially helps to mitigate against the threat of searching by abstract only.

Secondly, the field of AIEd, learning analytics, and related data-driven domains continues to evolve, and so do the terms used to describe interventions in these fields (e.g., data analytics, learning analytics, classroom analytics might be used interchangeably). For example, Rabbany et al. (2012), Farrell et al. (2013), and Lam et al. (2019) were not caught by our search because they simply referred to their interventions as “toolkits” and “online platforms” in the abstract (though, they would have been excluded at full-text review, regardless, because they do not evaluate the effectiveness of the intervention on bias/fairness/equity). As such, we may not have captured all relevant terms used to describe data-driven technologies, thus excluding relevant evidence.

Ultimately, our results and proposed framework for debiasing with data-driven technologies is based on the findings of only one existing intervention across two studies, which limits their generalisability. Resultingly, this paper is much more qualitative than other systematic reviews and meta-analyses that benefit from larger datasets. But like other *qualitative* research, we took precautions to enhance the *transferability* of our results, by including “thick descriptions” (Guba and Lincoln, 1982) of the included studies; in this way, we hope that readers take care to compare the EQUIP context to their own and assess in which ways the results can inform their own research. Furthermore, we also included details from several full-text items that did not meet the eligibility criteria, which should help readers understand the general landscape of research related to this topic. Finally, in the construction of our framework, we draw insights not only from our included studies, but also from other literature that we found to be relevant, to build a proposal for a way forward. Ultimately, more research needs to be conducted on how data-driven interventions impact teachers' biases to draw any generalisable conclusions about how to design data-driven technologies that promote debiasing; we hope that our framework can help jump-start this research and we expect it to evolve as more evidence comes to light. We also make our search and analysis process as transparent as possible to allow auditability and reliable repetition if needed and argue that our framework can provide researchers and practitioners with these potentially transferable and auditable findings; and we do not argue for generalisability. As a starting point, we hope that this framework and review acts as a call-to-action and discussion-point for future design, development, and evaluation of data-driven technologies to address implicit biases in education and beyond.

## Conclusion

One of the core arguments for the use of data-driven technologies in education is that they enable teachers to make fair and objective decisions in the classroom (Lameras and Arnab, 2022; Uttamchandani and Quick, 2022; Williamson and Kizilcec, 2022). However, the findings of this systematic review indicate that this argument has not been evidenced—both in the fields of AIEd and data-driven technologies more broadly—and, contrastingly, has highlighted that data-driven technologies have the potential to perpetuate implicit biases and inequitable teaching practices. Whilst there is much research regarding the responsible nature of data-driven technologies that deals with bias from a technical perspective (Floridi and Cowls, 2019; Baker and Hawn, 2021), responsibility from the perspective of how these technologies impact biases in end-users has largely been ignored, despite its role as a core justification for the creation of data-driven technologies. We propose that now is the time to “get real” and start explicitly and purposefully designing decision-support interventions both to mitigate against worsening biases and to actively challenge existing biases of teachers, to increase the responsibility of these tools from a human perspective. To support this call to action, we offer directions for the design, development, and evaluation of future data-driven technologies to support teachers' decision-making and extend Larrick (2004)'s motivational, cognitive, and technological debiasing strategies framework to the context of data-driven decision-making systems. We hope that this acts as a source of inspiration and motivation for considering the design of data-driven tools in a more holistic way, rather than only focusing on techno-centric solutions to address bias in teacher decision making.

## Data availability statement

Publicly available datasets were analyzed in this study. This data can be found here: https://osf.io/d49p8/?viewonly=f440a19292774cf8bf4873058b1bcb13&lt.

## Author contributions

AG performed the searches of the databases and led the data collection and write-up of the manuscript. SR was second coder in the data collection/extraction and contributed to the writing of the manuscript. MC and MM contributed to the writing of the manuscript, framing of the research, and secured the funding. All authors contributed to the article and approved the submitted version.

## Funding

This research is partially funded by UK Research and Innovation (EP/P025544/2) and UCL IOE Faculty of Education and Society strategic seed funding.

## Conflict of interest

The authors declare that the research was conducted in the absence of any commercial or financial relationships that could be construed as a potential conflict of interest.

## Publisher's note

All claims expressed in this article are solely those of the authors and do not necessarily represent those of their affiliated organizations, or those of the publisher, the editors and the reviewers. Any product that may be evaluated in this article, or claim that may be made by its manufacturer, is not guaranteed or endorsed by the publisher.
